# A Second Life for eHealth: Prospects for the Use of 3-D Virtual Worlds in Clinical Psychology

**DOI:** 10.2196/jmir.1029

**Published:** 2008-08-05

**Authors:** Alessandra Gorini, Andrea Gaggioli, Cinzia Vigna, Giuseppe Riva

**Affiliations:** ^3^Psychology DepartmentCatholic University of MilanMilanItaly; ^2^Research Institute Brain and BehaviourMaastricht UniversityMaastrichtThe Netherlands; ^1^Applied Technology for Neuro-Psychology LabIstituto Auxologico ItalianoMilanItaly

**Keywords:** 3-D virtual worlds, virtual reality, eHealth, p-health, Second Life

## Abstract

The aim of the present paper is to describe the role played by three-dimensional (3-D) virtual worlds in eHealth applications, addressing some potential advantages and issues related to the use of this emerging medium in clinical practice. Due to the enormous diffusion of the World Wide Web (WWW), telepsychology, and telehealth in general, have become accepted and validated methods for the treatment of many different health care concerns. The introduction of the Web 2.0 has facilitated the development of new forms of collaborative interaction between multiple users based on 3-D virtual worlds. This paper describes the development and implementation of a form of tailored immersive e-therapy called p-health whose key factor is interreality, that is, the creation of a hybrid augmented experience merging physical and virtual worlds. We suggest that compared with conventional telehealth applications such as emails, chat, and videoconferences, the interaction between real and 3-D virtual worlds may convey greater feelings of presence, facilitate the clinical communication process, positively influence group processes and cohesiveness in group-based therapies, and foster higher levels of interpersonal trust between therapists and patients. However, challenges related to the potentially addictive nature of such virtual worlds and questions related to privacy and personal safety will also be discussed.

## Introduction

Since the introduction of the Web 2.0 in 2004 [1], there has been a huge increase in the potential of Web applications, allowing users to create, modify, and share contents using multiple computers in various locations. The Web 2.0 is a read-write Web that facilitates social networking, collaboration, and participation between users [2,3]. One hugely successful application of the Web 2.0 is represented by three-dimensional (3-D) virtual worlds (eg, Second Life [4], There [5], and Active Worlds [6]). These computer-based, simulated environments are characterized by the simultaneous presence of multiple users who inhabit and interact via avatars within the same simulated space.The computer-simulated world typically appears similar to the real world, with real world rules such as gravity, topography, locomotion, real-time actions, and communication.Over the last few years, the number of virtual world users has increased dramatically, and today, Second Life, the largest 3-D online digital world, boasts some 12 million subscribers. 3-D virtual worlds can be considered as 3-D social networks, where people can collaborate to create and edit objects (like a collaborative 3-D wiki space) besides meeting each other and interacting with existing objects. Compared with the conventional Web 1.0 applications, virtual worlds offer novel ways to develop social skills; socialize and interact with other people via customizable, realistic, 3-D, fully textured, and animated avatars; attend and participate in live events like lectures and conferences; build communities, including learners’ communities and patient support groups; relax and visit new places; and browse document collections in 3-D virtual libraries. 3-D virtual reality (VR) worlds also show great potential for health purposes. In particular, Second Life currently features a number of medical and health education projects. By way of example, the Nutrition Game proposed by the Ohio University [7,8] simulates choices a user can make in various restaurants and informs the player about the health impacts of those choices. The Heart Murmur Sim [9,10] provides an educational virtual world for cardiac auscultation training that enables clinical students to tour a virtual clinic and test their skills at identifying the sounds of different types of heart murmurs. The Second Life Virtual Hallucinations Lab [11,12] aims to educate people about schizophrenic hallucinations. The Gene Pool [13] is an interactive genetics lab and learning area featuring simulated lab experiments, tutorials, and simple videos to enhance the learning experience. The Virtual Neurological Education Centre (VNEC) [14] demonstrates a virtual simulated online experience where people are able to actively expose themselves to the most common symptoms that a person suffering from a neurological disability may encounter, and the HealthInfo Island [15] is funded by the US National Library of Medicine (NLM) to provide consumer health information services. All of these virtual initiatives are mainly centered on the promotion of an innovative form of public health consisting of the diffusion of medical information and the education of therapists and patients [16].

The aim of the present paper is to introduce and discuss the use of 3-D virtual worlds for an innovative online health service called p-health. P-health provides personalized immersive e-therapy whose key factor is interreality [17], ie the creation of a hybrid-augmented experience merging the physical and virtual world. In p-health, the interreality experience is achieved through the following:

- an extended sense of presence [18-20]: P-health uses advanced simulations (3-D virtual worlds) to transform health guidelines and provisions into experience. In p-health, users do not receive abstract information but live meaningful experiences.
- an extended sense of community (social presence): P-health uses hybrid social interaction and dynamics of group sessions to provide each user with targeted—but also anonymous, if required—social support in both the physical and virtual world.
- real-time feedback between the physical and virtual worlds: P-health uses bio and activity sensors and devices (eg, PDAs, mobile phones) to track both the behavior and the health status of the user in real time and to provide targeted suggestions and guidelines. The feedback activity is twofold: (1) behavior in physical world influences the experience in the virtual one (eg, if I eat too much and I do not exercise, my avatar will become fatter), and (2) behavior in the virtual world influences the experience in the real one (eg, if I participate in the virtual support group, I can exchange SMS messages with the other participants during the day).

Our hypothesis is that the introduction of the p-health approach in eHealth services could extend the potential of the Web 2.0 and shared 3-D worlds to therapists and patients. To support this claim, the paper will describe how the use of avatars can improve social presence. Further, we will focus on the existing applications of 3-D worlds in clinical settings and address some ethical considerations and possible pitfalls of using 3-D worlds for therapeutic purposes. Finally, we will introduce a possible p-health scenario we are developing in Second Life for the treatment of addiction disorders.

## Psychological Features in Avatar-Based Interaction

The p-health approach suggests that providing remote patients with a feeling of social presence [21] plays a crucial role in improving therapeutic effectiveness. Through social presence, users experience a feeling of inhabiting a shared space with one or more others, and their awareness of mediation by technology recedes into the background [22]. Social presence requires participants to experience themselves as co-located and mutually aware of, responsive to, and responsible to one another [23]. As suggested by Casanueva and Blake [24], the sense of social presence consists of the belief that the other people in the virtual environment are real and really present and that the user and the others are part of a group and process.

We suggest that 3-D virtual worlds are able to convey strong feelings of social presence through avatar interaction, enhancing the feeling of togetherness of remote users who are connected through some form of telecommunication medium. Results of recent studies about avatar-based social interaction lend support to this hypothesis [25]. In their research, Bente and colleagues [26] measured social presence and interpersonal trust in avatar-based collaborative net communications, comparing this condition with face-to-face communication as well as with audio-based (phone) and text-based Web communication. The results from 48 participants showed that the level of co-presence was higher in avatar-based interactions than in phone or chat interactions. In a subsequent study, Bente and colleagues [27] investigated the experience of social presence as a relevant effect dimension of avatar-mediated Web communication. A total of 142 participants were randomly assigned to one of five possible communication settings: (1) text only, (2) audio only, (3) audio and video, (4) audio and low fidelity avatar, (5) audio and high fidelity avatar. Results revealed a significant difference between text and all other communication modes, indicating that audio, video, and avatar systems work similarly and better than text alone in creating the experience of social presence. However, according to the authors, avatar platforms offer new potentials to overcome many of the restrictions related to audio and video communication modes. In particular, they suggest that virtual worlds and avatars play a critical role in contextualizing social interaction and fostering the salience of nonverbal information by providing active filtering and contingency management systems as opposed to being just the virtual equivalents of a video conferencing system.

Other studies have suggested that even avatars with rather primitive expressive abilities may elicit strong emotional responses in users sharing a collaborative virtual environment. Experiments have shown that the avatar can readily take on a personal role, increasing the sense of community feeling and becoming a genuine representation of the underlying individual, not only visually, but also within a social context [28]. Moreover, Yee and colleagues [29] investigated whether norms about social space in the real world map onto how avatars act in relation to each other in virtual space. In an observational study, the authors collected data from avatars in order to explore whether social norms of gender, interpersonal distance (IPD), and eye gaze transfer into virtual environments even though the modality of movement is entirely different. They found that, as in the real world, male-male dyads tend to stand further apart and look at each other much less than female-female dyads: (1) male-male dyads have larger IPDs than female-female dyads, (2) male-male dyads maintain less eye contact than female-female dyads, and (3) decreases in IPD are compensated with gaze avoidance. In summary, all these preliminary findings suggest that avatar-based interaction in virtual worlds may have the potential to enrich the level of emotional connections and social presence conveyed by conventional telehealth tools, such as Internet, videoconferencing, email, and telephone.

From a clinical perspective, the advantages presented by the 3-D, avatar-based interactions serve to facilitate the communication process between therapists and patients, to positively influence group cohesiveness in group-based therapies and to create greater levels of interpersonal trust, which is a fundamental requirement to establish a successful therapeutic alliance.

## 3-D Virtual Worlds As a Support Tool for Psychological Interventions

The strong sense of presence and social connection elicited by avatar interaction suggests two possible clinical applications of 3-D virtual worlds. The first regards the potential to provide VR-based treatments within the online virtual worlds, and the second regards the creation of online virtual communities of patients.

### 3-D Virtual Worlds and Virtual Reality Exposure Therapy

In recent years, a number of studies have suggested the efficacy of VR exposure therapy in the diagnosis and treatment of various psychological disorders. Positive results have been obtained in the treatment of specific phobias (in particular, aviophobia, acrophobia, fear of driving, claustrophobia, and arachnophobia), eating disorders, social anxiety disorders, sexual disorders, posttraumatic stress disorder, and panic disorder with or without agoraphobia [30,31].

During the VR exposure, the patient is immersed in a virtual environment containing the critical stimulus. This procedure, which has been shown to be at least as effective as traditional techniques in reducing phobic symptoms [32], presents some practical advantages offered by the use of VR technology. As stimuli are computer generated, the therapist has full control over their intensity, and the risk of unpredictable effects is significantly lower than in vivo exposure since subjects have the opportunity to explore threatening aspects of reality in a safe environment where consequences are not real [33]. Further, virtual exposure provides an opportunity to present the patient with realistic 3-D visualization of the feared situation, which is more effective than imagination, especially when the patient is unable to recreate the critical scenarios because of pathological avoidance of problematic memories, as is often the case in posttraumatic stress disorder [34]. When used in combination with specific instruments, the VR exposure has the added advantage of allowing therapists to record different psycho-physiological parameters before, during, and after exposure to the feared stimuli in order to obtain objective measures of the individual modifications.

3-D virtual worlds appear to have much to offer to exposure therapy of this kind. The therapist and patient share the same online virtual space and, in this way, the therapist can accompany the patient through a particular threatening experience just by logging onto a specific website and adopting a preferred avatar. Interaction can be modified on the basis of therapeutic needs. In the case of social phobia, for example, after practising with the therapist within a closed environment (ie, the therapist’s virtual office), the patient can be taken to a virtual world populated by other avatars and asked to initiate a conversation and obtain feedback from them in real-time audio through the use of a microphone. Similarly, patients with agoraphobia can be exposed to a variety of unfamiliar worlds different from those the clinician can provide in an office setting. Patients suffering from addiction disorders (eg, drug abuse, pathological gambling, food craving) can be exposed to specific kinds of dangerous stimuli without running the risk of “succumbing to temptation” [35].

### 3-D Virtual Worlds for Creating Virtual Communities of Patients

3-D virtual worlds may have the potential to bring several innovative features to virtual patient communities by providing mediated environments with appropriate social, nonverbal, and contextual information that previous Web applications (Web 1.0) were unable to convey. Winkelman and Choo [36] surmised that patients with chronic diseases possess a particular tacit knowledge gleaned from their personal experience of illness and experientially acquired by having to cope with the daily challenges and needs posed by a chronic disease. These needs include information on the disease, treatment side effects, treatment plans, professional contacts, as well as supportive information for family and friends. According to the authors, if this tacit knowledge can be shared or socialized through a program, tool, or medium, a patient’s sense of self-efficacy can improve, thereby positively affecting health outcomes as well as social functioning. This approach argues for a shift in the role of chronic disease patients from external consumers of health care services to a community of practice of internal customers. Introduced by Wenger (1998), communities of practice are social constructs that bring learning into lived experience of participation in the world [37]. They are defined as self-organizing, informal groups whose members work together toward common goals, face common needs, share best practices, and have a common identity. Drawing on these concepts, Winkelman and Choo [36] suggest that with the implicit support of health care organizations, patients can benefit from gaining access to the expertise of peers by integrating knowledge gained from the experiences of living with chronic disease into their self-management. In particular, they claim that virtual patient communities can become effective tools of communication if (1) members have common interests, needs, goals, as well as an aspiration for mutual communication and the furthering of relationships, and (2) they are able to supplement already existing face-to-face communication opportunities. Even in this case, the possibility to share specific virtual environments from different parts of the world and to interact via customizable avatars can presumably facilitate the development and the diffusion of online communities of practice allowing an efficient exchange of medical and experiential information between patients and experts.

## Existing Therapeutic Applications in Second Life

In this section we will briefly explore some of the Second Life virtual environments specifically created for therapeutic purposes. Inspired by the therapeutic success obtained with different kinds of virtual treatments [38,39], and taking advantage of the potential of the Second Life platform, Brain Talk Industries, the largest nonprofit organization in the world dedicated to providing online communities for patients and caregivers dealing with neurological issues, has created Brigadoon, a private island in Second Life specifically designed for patients with Asperger’s syndrome. Brigadoon aims at providing an ideal place for people with a form of high-functioning autism, characterized by enormous difficulties in social interactions, to develop their social skills by interacting with other people dealing with the same problems [40]. After their initial experiences inside Brigadoon, many patients began venturing into Second Life proper and mixing with nonautistic people. Some of them are now active participants in other communities, including two autistic women who have formed "the autistic liberation front," a Second Life space where autistic people can organize, educate, and advocate for themselves [41-43]. A similar aim underlies the creation of Live2Give [44]*,* a Second Life island dedicated to people affected by cerebral palsy. Like Brigadoon, this virtual place brings people together, giving them the possibility to help each other cope with their common struggles. According to Lester, the experience appears to be empowering and revolutionizes the way the users feel about themselves and the part they have to play in the world [45]. Similarly, a British organization called ARCI has developed a virtual environment in Second Life to help abused children learn important life skills. The children enter the virtual world to learn to socialize and work as a team and to learn essential computer skills [46]. A very interesting therapeutic experience related to Second Life is described by Roberto Salvatierra, a medical student suffering from agoraphobia. Within Second Life, he created an avatar that closely resembled his own real-life appearance. By seeing himself in a simulated 3-D environment, Roberto felt he could become more comfortable with unfamiliar open spaces and this was exactly what happened. Thanks to his personal positive experience he decided to set up an in-world group called the "Agoraphobia Support Group," which he hopes other people with agoraphobia will join to discuss their shared difficulties [47,48].

These examples show how 3-D online virtual worlds can provide a richer variety of tools than email or typing text onto bulletin boards, including the opportunity to build new customized environments, create avatars, interact with others without revealing one’s real identity (ie, the real physical disabilities one has in the real world), and communicate with people in a way that more closely resembles face-to-face meetings. Moreover, the possibility to buy gestures—animations of avatars making faces—enriches the way in which users can communicate and represent themselves in these experiential virtual worlds. So, even if the main aim of these virtual online communities is to support rather than treat patients, their success proves the potential of 3-D virtual worlds to become very useful tools for an innovative form of eHealth dedicated to patients with mental illness [16,49].

Despite the positive data we have presented, the use of the Internet to provide mental health services is controversial, and, in the ongoing debate about the value and ethics of therapeutic virtual environments, there are proponents at both extremes. Some conceive of technology as means to a bright future where anyone's emotional needs can be instantaneously addressed; others are obstinately opposed to the use of distance psychology for any kind of intervention. In our view, virtual therapy is most effective when it is used as an adjunct to traditional therapy or as part of an aftercare plan. For these reasons, we advise against any kind of therapy being practised exclusively on the Web because of its supportive rather that exhaustive nature. This point must be made clear to online therapy providers and the general public.

## Ethical Considerations and Important Caveats in the Use of 3-D Virtual Worlds for eHealth

Although the therapeutic potential of 3-D virtual worlds is quite promising, there are challenges associated with an approach of this kind that need to be addressed. In fact, if it is true that people can explore threatening aspects of reality in a safe environment, it also is true that if the use of online worlds becomes excessive, there is a risk that it will prevent people from forming meaningful real-world relationships. In fact, as observed by Allison et al [33], an increased substitution of cyberspace-based relationships at the expense of face-to-face interaction may create a developmental double-edged sword. In the case of socially anxious patients, for example, the Internet is useful to modify peer group interactions, while it does little to foster the development of genuine intimacy. When exposing patients to virtual environments, therapists should consider the risk of Web addiction and encourage patients to participate in real-life social interaction as much as possible.

Another critical point regards anonymity: the chance to remain anonymous offers a less intimidating opportunity for social interaction and psychological reflection and would allow more people to discreetly seek help on their own. On the other side, anonymity represents a significant risk for patients and therapists. The computer-based interface does not guarantee that the person on the other side of the screen is really who we expect, and anybody can enter the virtual environment and interact with patients, producing negative effects on their experience and introducing uncontrollable and disturbance variables in the environment. These aspects can be overcome, for example, by creating private servers specific for controlled environments designed and dedicated to therapy and using protection codes personally given by the therapist to the patients.

Regarding the therapists, they need to first conduct self-assessment and then enhance their knowledge and skills in using these alternative forms of therapy [50] since the provision of eHealth services is not simply a click of the mouse [51].

Besides the previous more clinical considerations, there are some very challenging issues that need to be resolved to ensure the safe and ethical use of eHealth in general. These include complex and interrelated questions of security, confidentiality, and privacy; licensure requirements; competency; standards of care; and reimbursement that must be considered by practitioners, researchers, consumers, health care organizations, managed care companies, and federal and state legislatures [52].

The American Psychological Association (APA) has published a statement entitled "Services by Telephone, Teleconferencing, and Internet" [53]. This statement stipulates that in the absence of specific telehealth standards, psychologists must take reasonable steps to ensure competence in providing services and to protect patients, clients, and research participants from harm. The APA is also developing recommendations for the board regarding ethical, legal, and clinical concerns related to the practice of telehealth, with the aim of providing practitioners with information about electronic activities. While conducting interventions via telehealth applications, patients may believe that the Internet sessions are secure and completely private and confidential. To safeguard against a breach in confidentiality, therapists and clinicians should fully inform patients of the limits of confidentiality associated with telehealth and other forms of telecommunications. In sum, the use of 3-D virtual worlds as an advanced form of eHealth holds great promise as long as their limitations and associated risks are taken into consideration as well.

## The Use of 3-D Virtual Worlds in Clinical Practice

In the Introduction, we presented p-health as a possible new paradigm for eHealth. From a technological viewpoint, a possible p-health scenario would be based on the following three technologies: 3-D virtual worlds, bio and activity sensors, and personal digital assistants (PDAs) and/or mobile phones. Each will be considered in turn below.

### 3-D Virtual Worlds

As we have discussed previously, 3-D virtual worlds enable their users to interact with each other through motional avatars. Residents can explore the world, meet other users, socialize, participate in individual and group activities, and buy items (virtual property) and services from one another.

### Bio and Activity Sensors (Connection from the Real World to the Virtual One)

Typically 3-D virtual worlds are closed worlds and in no way reflect the real activity and status of the users. In p-health, bio and activity sensors are used to track the health status of users and to influence their experiences in the virtual world (avatar, activity, and access). The link between real and virtual worlds would be in real time, allowing the development of advanced biofeedback settings, but would also ensure health tracking even in situations where an Internet connection is unavailable.

### PDAs and/or Mobile Phones (Connection from the Virtual World to the Real One)

In p-health, the social and individual user activities in the virtual world have a direct link with his or her life through a PDA and/or mobile phone. This link is at three levels:

1. follow-up: It is possible to assess and improve the outcome of the virtual experience through the PDA/mobile phone, eventually also using information from the bio and activity sensors.
2. training and homework: Due to the advanced graphic and communication capabilities now available on PDAs/smart phones, they can be used as simulation devices to facilitate the real-world transfer of knowledge acquired in the virtual world.
3. community: The social links created in the virtual world can be continued in the real one even without revealing the real identity of the user; for example, I can send an SMS to a virtual friend in my own real context to ask for support.

It is our view that in p-health the creation of a direct link between the real world experience and the virtual one would serve to improve the accessibility of relevant information, the real-time monitoring of relevant health parameters, the motivation for change, the transfer of acquired knowledge in the real world, the social support, and the availability of anonymous expert guidance.

## A Possible Scenario: Addiction

P-health is an approach to health that, in theory, can be used for any kind of health concern. However, to discuss its feasibility, we decided to identify one possible area of intervention: addiction. The term *addiction* indicates a recurring by an individual to engage in some specific activity despite harmful consequences to the individual's health, mental state, or social life. The term was originally reserved for drug addictions, but it is now also applied to other compulsions such as pathological gambling, compulsive overeating, alcoholism, and so on. Addiction is a disease [54], a state of physiological or psychological dependence on something manifesting as a condition in which medically significant symptoms liable to have a damaging effect are present. Treatment of dependency is usually conducted by a wide range of medical and allied professionals, including addiction medicine specialists, psychiatrists, appropriately trained nurses, social workers, and counselors, and is focused on the individual's ultimate decision to pursue an alternate course of action. Behavioral treatments usually involve the planning of specific ways to avoid the addictive stimulus and therapeutic interventions intended to help a patient learn healthier ways to find satisfaction.

Literature on behavioral analysis and behavioral psychology shows that behavioral therapy, community reinforcement approaches, cue exposure therapy, social skills training, and contingency management strategies are useful approaches for the treatment of addiction [55]. Following these indications, we are developing Eureka [56], a Second Life island for addiction prevention and treatment. Eureka is a virtual immersive environment organized around three different but interconnected areas: the Learning area, the Community area, and the Experience area*.*

**Figure 1:**
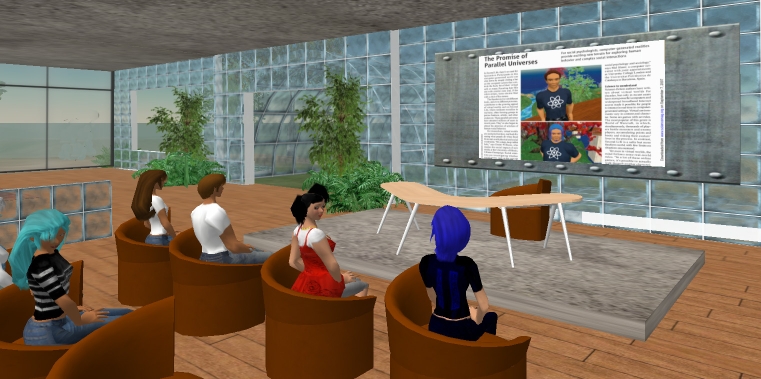
A screenshot from the Learning area [56]

The goal of the Learning area is to use motivation provided by virtual worlds to teach users about how to improve their living habits. The Learning area is organized around different learning areas (Figure 1), both without and with teachers (classes). In this area, users learn how to manage daily choices and activities, acquire general and specific information about addiction, and get the information needed to succeed, with daily tips and expert ideas.

**Figure 2:**
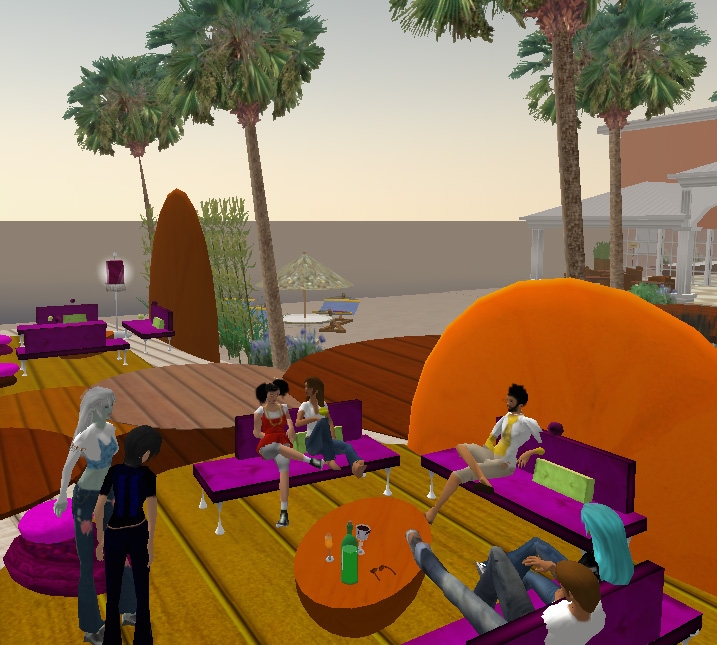
A screenshot from the Community area

The goal of the Community area is to use the strength of virtual communities to provide real-life insights aimed at improving living habits. The Community area is organized around different zones (Figure 2) in which users discuss and share experiences among themselves with or without the supervision of an expert (physician, psychologist, nutritionist, etc).

In the Learning and Community areas, users enjoy support and guidance, learn how to make wise choices and live healthily, and benefit from the exchange of practical experiences and tips from other users.

**Figure 3:**
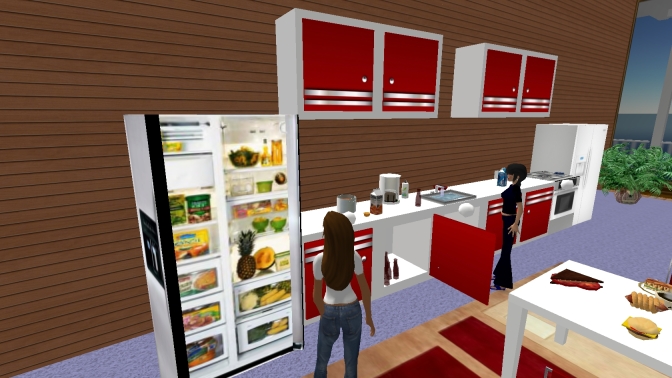
A screenshot from the Experience area (In particular, the kitchen has been created for people affected by food craving.)

The goal of the Experience area is to use the feeling of presence provided by the virtual experience to practise both emotional and relational management and general decision-making and problem-solving skills. This area includes different zones (Figure 3) presenting critical situations related to the maintenance and relapse mechanisms (Mall, Supermarket, Pub, Restaurant, Kitchen, etc). Each of these environments is experienced only under supervision.

In all three of these areas, the user is helped to develop specific strategies for avoiding and/or coping with their problems. After the experience, the coach explores the patient’s understanding of what happened in the virtual experience and the specific reactions—emotional and behavioral—to the different situations experienced. If needed, some new strategies for coping with the situations are presented and discussed. In all three areas, type and intensity of care will vary depending on the type of intervention (eg, prevention vs treatment).

In our vision, Eureka could be an interesting starting point to test the efficacy of online virtual worlds in the prevention and treatment of different psychological disorders.

## Conclusions

This paper addresses a broad and emerging idea in the field of eHealth: the use of 3-D virtual worlds for online mental health applications. As we have recently discussed elsewhere [57], 3-D online worlds have become not only fertile ground for psychologists exploring human behavior [58], but they are also starting to play an emergent role in health services. Why should this be so? Compared with traditional telehealth systems (videoconferencing, email, telephone, Web 1.0 applications, etc) and other available technologies (eg, CD or DVD), 3-D virtual worlds provide users with a more immersive and socially interactive experience, as well as a feeling of embodiment that has the potential to facilitate the clinical communication process and to positively influence group interaction and cohesiveness in group-based therapies. Moreover, unlike the available VR software (see, for example, NeuroVR [59]), 3-D virtual worlds, being Internet-based applications, can be used by different people from different places without physical limitations.

Although this new medium has the potential to improve existing eHealth applications, there are several challenges that need to be addressed. First, more basic psychological research is needed in order to gain a clearer understanding of psychological, communicative, and interpersonal aspects of avatar-based interactions and of the differences between this and other interaction modes. Second, to date, there is scant encouraging data coming from traditional telepsychology applications [60-63] and online communities [39,44] and no experimental or controlled data about the therapeutic effectiveness of online virtual worlds in patients with mental health disorders. Third, 3-D virtual worlds were not created with clinical purposes in mind. This means that clinicians and researchers have to create specific and protected environments to meet their clinical needs as well as the needs of patients. Further, as for any kind of eHealth system, it is important to define international guidelines for the development of 3-D virtual world–based clinical applications in order to reduce the risk of abuse and to guarantee appropriate levels of privacy. Finally, online virtual worlds have open access, meaning that it may be difficult to create safe therapeutic environments in which patients can interact with therapists without external interferences and with privacy protection. Also, cost issues should not be overlooked. The vast majority of virtual worlds have high subscription costs, which may be too expensive for private therapists; in February 2008, the price for an island in Second Life was US $1675 plus a US $295 monthly fee. Finally, most online worlds provide users with building tools (editors) that are not easy to use for nonexperts as they often require the user to learn script-based programming languages.

In conclusion, despite technical, ethical, and economic issues, we suggest that 3-D virtual worlds, used as an adjunct to face-to-face settings, may represent a valid opportunity for the future developments in eHealth. Our hope is that the present paper will stimulate a discussion within the research community about the potential, the limitations, and the risks that this emerging medium offers for cybertherapy applications.

## Abbreviations

3-D: three dimensional
IPD: interpersonal distance
PDA: personal digital assistant
VR: virtual reality
